# Prophages and Past Prophage-Host Interactions Revealed by CRISPR Spacer Content in a Fish Pathogen

**DOI:** 10.3390/microorganisms8121919

**Published:** 2020-12-02

**Authors:** Elina Laanto, Janne J. Ravantti, Lotta-Riina Sundberg

**Affiliations:** 1 Molecular and Integrative Biosciences Research Programme, Faculty of Biological and Environmental Sciences, University of Helsinki, 00014 Helsinki, Finland; janne.ravantti@helsinki.fi; 2 Department of Biological and Environmental Science and Nanoscience Center, University of Jyvaskyla, 40014 Jyvaskyla, Finland; lotta-riina.sundberg@jyu.fi

**Keywords:** Bacteroidetes, CRISPR, *Flavobacterium columnare*, genome, prophage

## Abstract

The role of prophages in the evolution, diversification, or virulence of the fish pathogen *Flavobacterium columnare* has not been studied thus far. Here, we describe a functional spontaneously inducing prophage fF4 from the *F. columnare* type strain ATCC 23463, which is not detectable with commonly used prophage search methods. We show that this prophage type has a global distribution and is present in strains isolated from Finland, Thailand, Japan, and North America. The virions of fF4 are myoviruses with contractile tails and infect only bacterial strains originating from Northern Finland. The fF4 resembles transposable phages by similar genome organization and several gene orthologs. Additional bioinformatic analyses reveal several species in the phylum Bacteroidetes that host a similar type of putative prophage, including bacteria that are important animal and human pathogens. Furthermore, a survey of *F. columnare* Clustered Regularly Interspaced Short Palindromic Repeats (CRISPR) spacers indicate a shared evolutionary history between *F. columnare* strains and the fF4 phage, and another putative prophage in the *F. columnare* strain ATCC 49512, named p49512. First, CRISPR spacer content from the two CRISPR loci (types II-C and VI-B) of the fF4 lysogen *F. columnare* ATCC 23463 revealed a phage terminase protein-matching spacer in the VI-B locus. This spacer is also present in two Chinese *F. columnare* strains. Second, CRISPR analysis revealed four *F. columnare* strains that contain unique spacers targeting different regions of the putative prophage p49512 in the *F. columnare* strain ATCC 49512, despite the geographical distance or genomovar of the different strains. This suggests a common ancestry for the *F. columnare* prophages and different host strains.

## 1. Introduction

The variation between individual genomes within one bacterial species can be largely explained by the presence or absence of prophages. Furthermore, genome evolution in bacteria is greatly affected by prophages through horizontal transfer of genetic material [1,2]. Thus, prophages provide one of the most exciting resources for phage–host interaction studies. Lysogeny (i.e., the state where a phage genome is either integrated into the host’s chromosome (e.g., phage lambda) [3] or exists an extrachromosomal state (e.g., phage P1) [4]) can serve as a hideout for the phage during unfavorable conditions. On the other hand, prophages can provide benefits for their bacterial hosts in multiple ways; for example, increasing the capacity to cause disease by providing toxins or adhesion factors (see [5] for a review). Phages integrate to a few conserved sites and minimize the negative effect of integration to the host genome [6]. In active lysogeny, however, the phage can act as a bacterial gene regulator [7]. Several model systems have revealed the molecular details of the various regulatory systems needed for the establishment of the lysogenic cycle and, on the contrary, how that stable relationship is interrupted, leading to excision of the phage genome and induction of the lytic life cycle. It is likely that when new phage–host pairs are studied, new variations will be identified.

There is an association between lysogeny and bacterial genetic and life history traits; therefore, in some bacterial clades, prophages are scarce [8]. However, sequences of viral origin can occupy up to one-fifth of the total bacterial genome length [9]. Metavirome analyses suggest that the percentage of unknown genes in phages is high [10]. Indeed, phage genomes make up a large part of the genetic dark matter and most of the genes in phage genomes have remained annotated with unknown functions, especially in cases of lesser-known phage–bacterium pairs. Furthermore, while the amount of genomic data is rapidly increasing, both the lack of prophages characterized in detail and the lack of functional data on phage genes hinders the identification of unknown prophages from bacterial genomes.

*Flavobacterium* (phylum Bacteroidetes) species are ubiquitous, especially in aquatic environments [11,12]. Some of the species are also important aquaculture pathogens, such as *F. columnare* [13]. Although the 16S rRNA gene suggests that known isolates of *F. columnare* fall into only four genomic groups [14,15], whole-genome analysis has suggested higher genetic diversity [16]. There are approximately 20 sequenced *F. columnare* genomes (complete and in multiple scaffolds in GenBank), which allows the detection of prophages in these strains.

Lytic phages infecting *F. columnare* have been isolated previously [17] and their interaction with the host bacterium suggests them to be suitable for phage therapy [18,19]. However, the role of prophages in the evolution and diversification of the host or in the virulence of *F. columnare* has not been studied. This gap in knowledge mostly arises due to the lack of genomic and experimental data on the putative prophages within this species. Clustered Regularly Interspaced Short Palindromic Repeats (CRISPR) form a genetic memory of previous phage infections [20], and CRISPR and CRISPR-associated (Cas) genes have been identified in *F. columnare* [16,21]. Combining CRISPR–Cas and prophage data could aid in elucidating past phage–host interactions in *F. columnare*. The bacterium has two functional loci: Type II-C with Cas9 and Type VI-B with Cas13b, which both acquire phage-targeting spacers, causing selection in the phage population [21,22].

Here, we characterized a functional prophage fF4, induced from the *F. columnare* type strain ATCC 23463, originally isolated from diseased fish in the United States in the 1950s [23]. The bacterial culture supernatant produced plaques only on strains originating from Northern Finland. We sequenced the phage genome and studied the morphology of the phage particles. The genomic data enabled the identification of several similar prophages in the available genome sequences of *F. columnare* and also in several other species belonging to phylum Bacteroidetes. In addition, a unique prophage area in another *F. columnare* type strain ATCC 49512 was investigated. Finally, using CRISPR spacer sequences and prophage elements, we could identify past interactions between different strains of *F. columnare* and prophages.

## 2. Materials and Methods

### 2.1. Bacteria and Phage Used in the Study

*F. columnare* strain ATCC 23463 (NCIMB 2248^T^) was isolated from salmon kidney in 1955 from the United States [23]. The strain carries a prophage, which spontaneously induces into the supernatant. Initially, phage plaque was isolated from the supernatant of ATCC 23463, spotted on the soft agar lawn of *F. columnare* strain C4 (a colony morphology variant of strain C1 [24]), and named fF4. C4 was used as a host for all phage propagation at room temperature in Shieh medium [25] using agar (1%) and soft agar (0.7%) for the top layer of agar.

### 2.2. Host Range of Phage fF4

Host range of the fF4 phage was tested against a collection of 107 bacterial strains (see Table S1) that included mostly *F. columnare* isolates, but also *F. psychrophilum*, *F. johnsoniae* and undefined *Flavobacterium* species. Spots (2 μL) of lysate and 10- and 100-fold dilutions were placed on the lawn of each strain and, after 2 days of incubation, individual plaques on the spot area were considered as positive for infection.

### 2.3. Phage fF4 Morphology

Phage fF4 lysate was prepared from double-layer agar plates with confluent lysis. Five milliliters of Shieh medium was placed on the plate and it was incubated at +8 °C for 1 h. The liquid was collected and sterile-filtered (pore size 0.22 μm, Thermo Fisher Scientific, Waltham, Massachusetts, USA). The lysate was then crude-purified using the ammonium acetate protocol described by Ackerman [20]. In brief, the lysate was centrifuged (Beckman coulter L-90K, 70 Ti-rotor, 25 000× *g*, 2 h, +4 °C), the pellet was resuspended again in 0.1 M ammonium acetate, and the procedure was repeated twice. The final pellet was resuspended in 0.02 M Tris–HCl (pH7.2) and a sample was placed on a copper-coated grid and labeled with 1% phosphotungstate acid (PTA) at pH 6.5. Imaging was performed with a Jeol JEM-1400 transmission electron microscope at 80 kV.

### 2.4. The Number of Free Phages in the Cultures of Strain ATCC 23463

To follow the number of free phages and to see whether temperature had an effect on the spontaneous induction of phage fF4, free phages were measured from strain ATCC 23463 grown at three temperatures for 24 h in total. Triplicate cultures [5 mL of over night (o/n) grown ATCC 23463 and 45 mL of Shieh medium] were incubated under constant shaking (120 rpm) at 18, 22, and 26 °C. Samples of 1 mL were taken from each replicate for optical density (OD) measurements (595 nm, Multiskan GO, Thermo Fisher Scientific, Waltham, MA, USA) at time points of 0, 1, 2, 4, 6, 11, and 24 h. The remainder of the 1-mL sample (400 μL) was centrifuged (10,000 rpm, 3 min) and the plaque-forming units per mL (PFU/mL) was determined from the supernatant using a double-layer agar method with *F. columnare* strain C4 as the host bacterium. The plate cultures were done at room temperature. In addition, in a separate experiment, the effect of mitomycin C on the number of free phages was investigated by adding mitomycin C in a final concentration of 0.5 μg/mL to a 50 mL culture of ATCC 23463 grown for 2 h at 22 °C. The OD and the number of free phages was measured after 4 and 22 h of induction from the mitomycin-C-treated culture and the control culture without addition as described above. This experiment did not include replicates.

### 2.5. Genome Analysis

The genomic DNA of fF4 was extracted using a method by Santos [26] with modifications. Briefly, 40 mM ZnCl_2_ was added to DNase- and RNase-treated sterile filtered lysate and incubated for 5 min. The pellet was resuspended to 1 mL of TES (0.1 M Tris, pH8; 0.1 M EDTA; 0.3 % SDS) buffer. After proteinase K treatment, the DNA was purified using the Genomic DNA extraction kit (Thermo Fisher Scientific, Waltham, MA, USA). Sequencing was performed with Roche 454 at LGC Genomics (Germany) using commercial paired-end 454 sequencing. All analyses were performed using GS De Novo Assembler version 2.9 (454 Life Sciences, Roche, Basel, Switzerland). The resulting three contigs were combined with PCR (primer sequences in Table S2) and Sanger sequencing. Open reading frames (ORFs) were predicted using Glimmer [27] and GeneMarkS [28], and searches against the database were done using BLAST [29] and HHPred [30]. Putative antiCRISPR proteins were searched using PaCRISPR [31]. Geneious version 7.1 was used as the software for all sequence analysis (see also below).

### 2.6. fF4-Like Prophages in Bacterial Genomes

The obtained genome sequence of fF4 was used to search for other prophages. The putative DDE-transposase of fF4 was searched against the public database using BlastP (May 2020). For the hits that were received, the corresponding genome regions were analyzed. Fingerprints (ORFs with the same putative function as were identified throughout the fF4 genome) were considered as positives and were analyzed in more detail. Easyfig [32] using BlastX was used to create genome alignments. In addition, two prophage search tools, PHASTER [33] and VirSorter [34], were used for the genomes and genome contigs where fF4-like regions were observed.

### 2.7. Analysis of Prophage-Matching CRISPR Spacers

The CRISPR spacers from the available complete *F. columnare* genomes (strain[genetic type]: accession number; ATCC 49512[I]:NC_016510.2; B185[I]:NZ_CP010992.1; 94-081[II]:NZ_CP013992.1; C#2[II]:NZ_CP015107.1; TC1691[I]:NZ_CP018912.1; Pf1[I]:NZ_CP016277, available at the NCBI https://www.ncbi.nlm.nih.gov/, downloaded in August 2019) [35,36,37,38,39] were searched using CRISPRCasfinder [40] that identifies both CRISPR arrays and Cas proteins. The direction of spacers (direction of transcription) was applied from [22], in the same direction as Cas 9 in II-C and same direction as Cas13b in VI-B. The repeat-spacer arrays of both CRISPR loci (Type II-C and Type VI-B) of strain ATCC 23463 were sequenced by Sanger sequencing of the PCR products as described earlier [21]. The obtained spacer sequences were searched against the fF4 genome. In addition, all detected spacers were aligned with the putative prophage region from ATCC 49512 that has been described earlier in [41] but not the complete length. The genomic region containing the phage was searched against the database using BlastN (May 2020). In addition, BlastP and HHPred were used to analyze the ORFs in the region. Here we analyzed the prophage to be longer and to extend to the complete unique sequence found only in strain ATCC 49512 (see below).

### 2.8. Data Availability

All sequences generated in this study have been deposited to the National Center for Biotechnology Information. The phage genome accession number is MN850656 and the CRISPR spacer regions of strain ATCC 23463 are under the accession numbers MN853160 (II-C locus) and MN853161 (VI-B locus).

## 3. Results

### 3.1. First Description of a Functional Prophage Isolate Infecting the Fish Pathogen F. columnare

**Figure 1:**
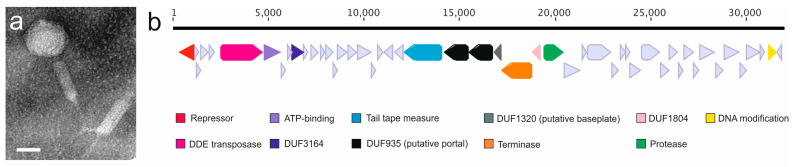
(**a**) Temperate phage fF4 of *Flavobacterium columnare* ATCC 23463 seen under a transmission electron microscope. Scale bar: 50 nm. (**b**) Genome organization of fF4 with predicted open reading frames (ORFs) and putative functions, when detected, indicated at the bottom.

Phage fF4 was initially isolated from the supernatant of type strain ATCC 23463. The supernatant produced plaques on four of the 107 tested bacterial strains: C1 (genetic group C and the morphotype derivant C4), E1 (genetic group E), B270 (genetic group C), and B436 (genetic group G) (Table S1). Under a transmission electron microscope (TEM), the phage particles displayed typical features of a myovirus: a head (~50 nm wide) attached to a contractile tail (~95 nm long) (Figure 1a).

### 3.2. Spontaneous Induction of Phage fF4

**Figure 2:**
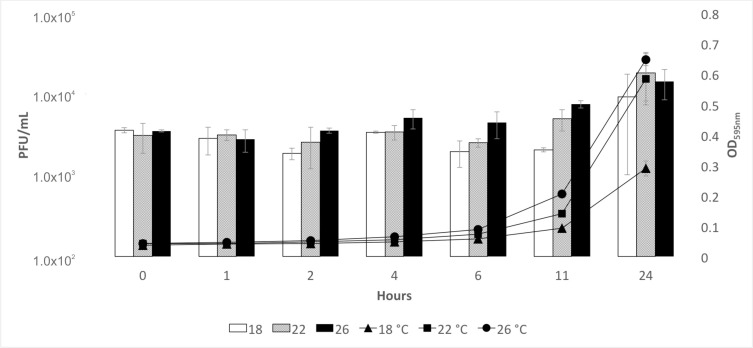
Mean and standard deviation of the amount of free fF4 phage as plaque-forming units per mL (PFU/mL, bars) and the optical density (595 nm, lines) from three replicates of *Flavobacterium columnare* strain ATCC 23463 grown in three temperatures (18, 22, and 26 °C) for 24 h.

The spontaneous induction of phages from ATCC 23463 was measured as number of free phages in cultures grown at three temperatures (Figure 2). The initial amount of free phage was approximately 3.4 × 10^3^ after the over night (o/n) grown culture was diluted to 50 mL. Titers (PFU/mL) remained rather stable throughout the experiment at 22 and 26 °C but decreased at 18 °C, while the optical density started to increase in all from the zero point. An increase in the PFU was seen in the latest time point. OD and PFU/mL were highest at 26 °C for the first 11 h but at the last time point (24 h), the number of free phages was highest at 22 °C (1.8 × 10^4^). According to a single experiment, phage fF4 seems to be inducible with mitomycin C. Four hours after induction, the PFU/mL of free phages was almost 10-fold higher (1.5 × 10^5^ PFU/mL) compared with the control culture (3.9 × 10^4^ PFU/mL). There was also a difference in OD (mitomycin-C-induced 0.034 compared with the control, 0.098). After 22 h, the number of free phages had decreased in both; however, the mitomycin-C-induced culture contained almost 50 times more free phages (6.6 × 10^4^ PFU/mL compared with the control with 1.4 × 10^3^ PFU/mL). Here, the OD of the mitomycin-C-induced culture (0.028) was considerably lower compared with the control (1.398).

### 3.3. fF4 Genome Displays Similarities to Transposable Phages

The genome sequencing of phage fF4 resulted in three contigs that were combined using PCR and Sanger sequencing. The genome is a 31,958-bp-long linear genome with a GC% of 36 with 50 open reading frames (ORFs), of which 10 are leftward-oriented (Figure 1b). However, sequencing of the genome ends was not successful. The presence of fF4 was confirmed in the original bacterial host genome (project accession NZ_PCMX00000000, in 181 contigs as of 5 February 2018, contig accession PCMX01000018), where an identical sequence with only one nucleotide difference was found.

BlastP and HHPred analysis of fF4 ORFs (Table S3) revealed orthologs of the conserved genes shared by the transposable phages recognized thus far: Mu gpA, B, H, F, G, and gp36 [42,43] (see below). Only GemA, which is also one of the conserved genes among the transposable phages classified under the Saltoviridae family [42,44], was not detected. In addition to the gene orthologs, there were similarities in genome organization. The HHPred search and conserved domain hits in BlastP suggest that the first ORF in fF4 encodes a repressor. In the phage Mu, the gene c codes for the repressor protein, which is needed for establishing the lysogenic cycle as well as for superinfection immunity [45]. Thus, ORF01 could serve as a superinfection immunity factor in *F. columnare*. In transposable phage Mu, “A” and “B” follow the repressor gene which functions in the transposition [46]; A codes for the transposase needed for integration and for replication of the phage genome during the lytic cycle; and B codes for an ATP-dependent DNA-binding protein. In fF4, the putative repressor is followed by four short ORFs for which no function was predicted and which are assigned as hypothetical proteins. These are then followed by ORFs that encode for a putative DDE-transposase (ORF06 in fF4, corresponding to A in Mu) and an ATP-binding protein (ORF07, B in Mu). Further downstream in ORF14 of fF4, HHPred indicated homology with Mu’s middle operon gene (mor), which is part of the semi-essential gene (SEE) module. In addition, the following module in Mu is for morphogenesis and contains the cell lysis gene. Similarly, in fF4, the ORF15 is homologous to T5 endolysin. Although we could not predict functions for the several of the next ORFs, ORFs encoding for putative structural proteins were identified: a head morphogenesis gene (ORF22); a tail tape measure gene (ORF23); a putative head morphogenesis gene (ORF24), which shared a conserved domain with Mu gene F (putatively involved in phage head protein synthesis); a DUF935, which is a putative portal gene and shares a conserved domain with the Mu portal protein gene H (ORF25); and a DUF1320, which shared a conserved domain with Mu (gp36) (ORF26). Furthermore, the similarity to the organization of Mu genome continued in the second last ORF (ORF49), with a putative function in DNA modification (homologous to several DNA adenine methylases in HHPred). In Mu, the last gene, named mom, codes for an unusual DNA modification protecting the phage from several host restriction endonucleases [47]. In addition, a terminase (ORF27) and a Clp protease (or a caudovirus prohead protease) (ORF29) were identified, which were followed by the putative major capsid gene (ORF30). ORF33 in fF4 encodes for a putative tail connector protein according to HHPred. The ORFs 37, 39, 40, and 43 are putative tail genes and ORF41 is a putative holin gene according to the searches. The anti-CRISPR search with PaCRISPR did not find putative anti-CRISPR proteins.

### 3.4. Genome Sequence of fF4 Revealed Putative Prophages in Fish Pathogenic Flavobacterium Species and in Several Other Species from the Phylum Bacteroidetes

**Figure 3:**
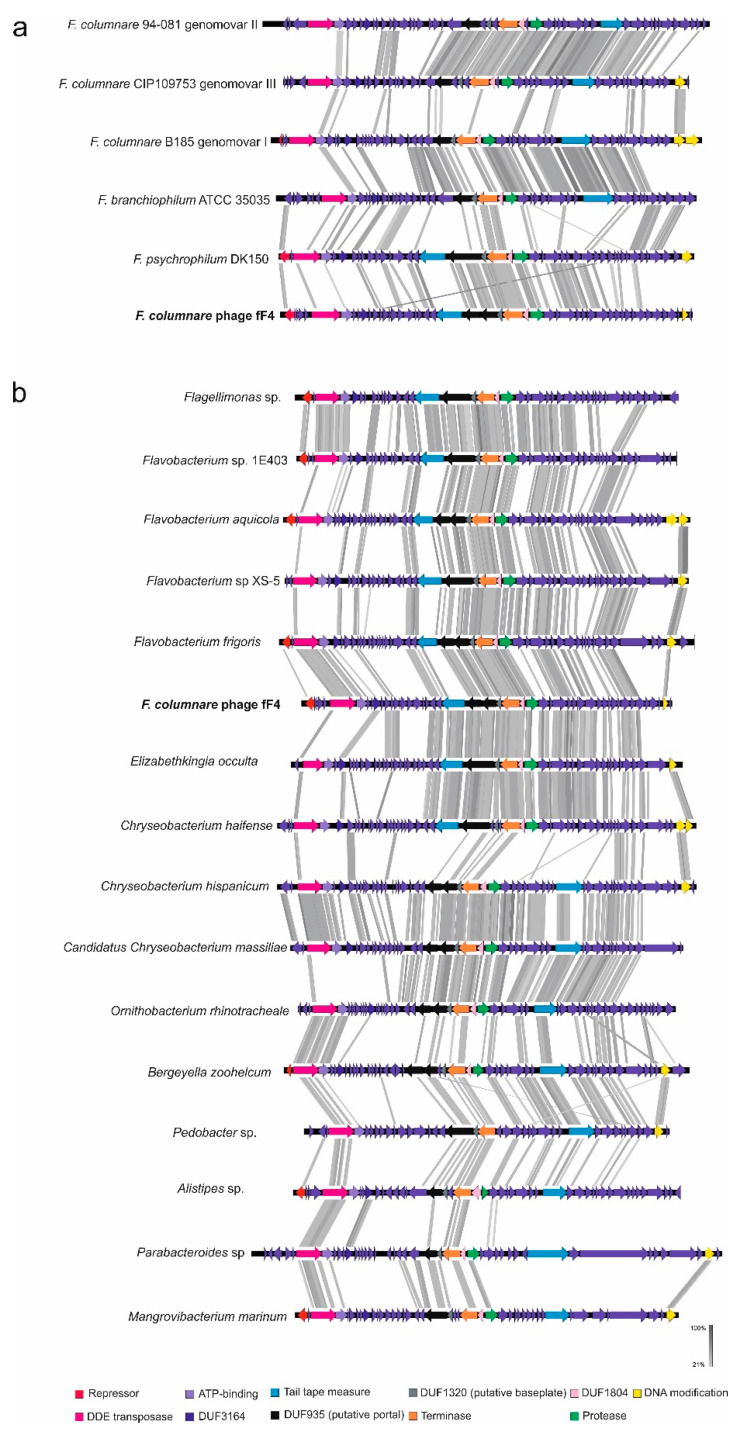
(**a**) Similarities between phage fF4 and the putative fF4-like prophages in the fish pathogen species of *Flavobacterium*. (**b**) The similarities between putative fF4-like prophages in the different species of Bacteroidetes (including phage fF4). Annotations are based on original annotations; some ORFs have been reannotated. ORFs with putative function found in the genomes are DDE-transposase with a following ATPase, near proximity to DUF3164, portal- and structure-related DUF935, DUF1320 followed by a terminase (annotation missing in most), a HTH transcriptional regulator (DUF1840), and a Clp protease (Caudovirus prohead protease). Tail tape measure protein was associated, in many cases, with other structural proteins a mentioned above or near to the right end of the putative prophage genome. One of the last or the last ORF is involved in DNA modification. In addition, other similarities were found (see Table S4 for details). Vertical blocks between sequences indicate regions of shared similarity shaded according to BlastX. Putative functions are marked with colors indicating the functions shown at the bottom.

We identified parts of fF4-related prophages in the genome contigs of three *F. columnare* strains isolated from Thailand (strains CF1, 1201, and 1215; Table 1). The longest match with the highest identity was with strain CF1 (Figure S1); it is possible that an active phage is also present in these three strains. In addition, BlastP was used to identify sequence homology with the putative DDE-transposase, and the genome regions from the corresponding hits were analyzed. Based on genome synteny and amino acid level similarities, we found fF4-type prophages from other *F. columnare* strains, as well as from other fish pathogenic *Flavobacterium* species (Figure 3a). A similar prophage (based on similar genes and organization) was found from the Finnish strain B185 (genetic Type I), from the Northern American strain 94-081 (genetic Type II), and the Japanese strain CIP109753 (genetic Type III) (see [14,15] for the genetic grouping), as well as from the fish pathogens *F. branchiophilum* ATCC 35035 and *F. psychrophilum* strain DK 150 (Figure 3a).

Furthermore, using the same approach (BlastP alignments of the fF4 DDE transposase and analysis of the corresponding bacterial genome areas), putative fF4-like prophages were detected in several different species, all from the phylum Bacteroidetes (Table 1 and Figure 3b). These included species such as *Flavobacterium* sp., *Chryserobacterium hispanicum*, *Flagellimonas* sp., *Pedobacter* sp., and *Elizabetkingia occulta* (Table 1). Similar prophage regions include (not all in the following order) DDE-transposase, followed by one or two ATPases and a DUF3164, (further downstream) phage tail tape measure protein; DUF935; DUF1320; terminase; and Clp protease (see Supplementary Table S4). In most of the genomes, one of the last ORFs was involved with DNA modification (such as DNA methyltransferase). PHASTER and VirSorter identified putative prophages from most of the genomes and contigs where fF4-like phages were found (Table S5). PHASTER was able to partly predict one of the putative fF4-prophages (in *Elizabethkingia occulta*), while VirSorter predicted the fF4-like prophage in most of the genomes/contigs; however, there were differences in the predicted region lengths (both shorter and longer predicted regions compared with the regions predicted by fF4- homologous phages).

### 3.5. Putative Prophage of the F. columnare Type Strain ATCC 49512 (pp49512)

**Figure 4:**
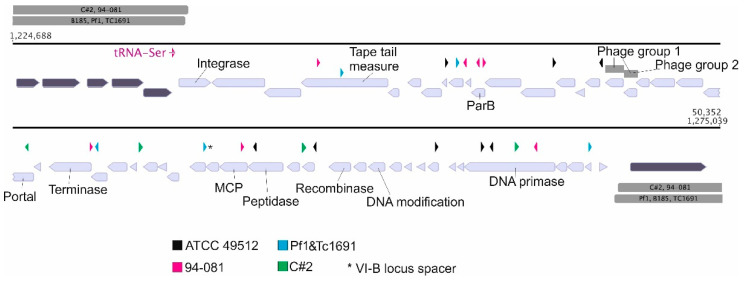
A putative prophage region pp49512 in the *F. columnare* strain ATCC 49512 with unique Clustered Regularly Interspaced Short Palindromic Repeats (CRISPR) spacers in different *F. columnare* strains that target the sequence in pp49512 (light colored ORFs) in the strain ATCC 49512 (ORFs surrounding the prophage are marked with darker color). All spacer hits from complete genome sequences with a maximum of two mismatches are shown with a color corresponding to the strain name indicated at the bottom (see also Supplementary Table S7). The direction of spacers was applied from [22]. Open reading frames (ORFs) with putative functions in the prophage are marked. The matching nucleotide sequences from other *F. columnare* genomes on both sides of the putative prophage area are shown in gray. Both sides have ~100% identity with strains TC1691, Pf1, B185, C#2, and 94-081. Sequences (~20 kbp area) that correspond to Finnish *F. columnare* phage isolates are also indicated (Phage group one: FCL-2, FCV-1, FCV-3, FCV-10, FCV-11, FCV-16, FCV-20, VK42, VK48. VK58; Phage group 2: V156, V157, V165, V181, V182). MCP stands for major capsid protein. See Supplementary Table S6 for details of the pp49512 ORFs.

In addition to fF4, we studied a putative prophage region in the *F. columnare* strain ATCC 49512, which we named pp49512 (putative prophage 49512). This region was selected for a more detailed analysis because many of the CRISPR spacers in other *F. columnare* strains targeted it (see below). This prophage region has been identified earlier using PHASTER, but not at full length (~19 kbp) [41] (in the prophage search in this study, PHASTER indicated the same region but ~26 kpb in length). We propose that the prophage is approximately 40 kbp long. This is suggested by the HHPred hits to major capsid protein and portal proteins; furthermore, a recombinase belonging to the tyrosine family is located near the 3′ end (Table S6). When aligned with the complete genomes of other *F. columnare* genomes (strains 94-081, C#2, TC1691, Pf1, and B185) the unique sequence (not found in other *F. columnare* strains) in this area extends longer (Figure 4). The matching sequences in the other *F. columnare* genomes (both ends of the prophage area) are far apart from the other end (for example, in the genome of *F. columnare* strain B185, the nucleotides are located at 90 kbp for one end and at 1.36 Mbp for the other end). There are also two sequence locations identical to the Finnish *F. columnare* phage isolates (Figure 4) [19,21], matching the ORFs annotated as hypothetical proteins in the left (e.g., in phage FCL-2 nucleotides 3285–4105) and the right side (43,049–43,743) of the approximately 47 kbp linear phage genomes. Interestingly, in pp49512, these areas are next to each other and both encode for a complete ORF. However, no function for these ORFs could be predicted.

### 3.6. One CRISPR Spacer in ATCC 23463 VI-B Loci Targets the Prophage fF4

The genome sequence of strain ATCC 23463 is in multiple scaffolds in GenBank and does not contain any CRISPR sequences. Here, the Type II-C and VI-B loci of the ATCC 23463 strain were Sanger sequenced to see whether the spacers matching the fF4 sequence (residing in this strain) could be found. A complete repeat spacer array was obtained for the VI-B locus (1040 nt), while the II-C locus remained in two pieces (775 and 762 nt). One spacer in the VI-B locus matches the putative terminase gene in fF4. Next, we surveyed the available CRISPR spacers from *F. columnare* strains in GenBank and checked the matches against fF4. Strains TC1691 and Pf1 both have the same terminase-matching spacer sequence in their VI-B loci (spacer sequence CTGTCTTACAAAGCAATCCAGTACGTGGA). In addition, Pf1 and TC1691 share an additional seven identical spacers in their VI-B locus as the type strain (of which two spacers are duplicates). One additional spacer has a two-nucleotide difference compared with the type strain.

### 3.7. Unique CRISPR Spacers in Several F. columnare Strains Match Putative Prophages in Strain ATCC 49512 (pp49512)

Several of the CRISPR spacers in ATCC 49512 target the putative prophage pp49512. Interestingly, other *F. columnare* strains also have unique spacers targeting the putative prophage genome region, based on BlastN (a unique spacer hit that was not shared with other strains and matched only the prophage) (Figure 4). These strains include representatives from genetic Types I and II: 94-081 (II) from the United States, C#2 (II) of unknown origin, and TC1691 and Pf1 from China (both I); the strain ATCC 49512 (I) itself originates from France. The analysis showed the VI-B locus in strains 94-081, ATCC 49512 and C#2 to contain spacers with less similarity and could not be therefore assigned to match the prophage region (see Table S7). Chinese strains Pf1 and TC169 contain identical II-C and IV-B loci and both loci have spacer sequences that target the prophage in ATCC 49512. These two strains were also the only ones to possess an identical spacer to the pp49512 prophage area in their VI-B locus.

The matching CRISPR spacers in ATCC 49512 are dispersed throughout the pp49512 prophage region (Figure 4). Two spacers from strain 94-081 match the putative ParB gene. In addition, spacers in strains 94-081 and Pf1/TC1691 target the putative tail tape measure protein. The putative DNA primase, possibly also part of the prophage, is targeted by two of the host spacers, as well as a spacer from C#2 and 94-081.

## 4. Discussion

Lysogeny is common in bacteria; genetic analyses indicate up to 20% of the bacterial genome can be of phage origin [9]. Although there is a risk that a temperate phage enters a lytic cycle and kills the host, bacteria tolerate temperate phages due to well-described advantages for the host. Phage-encoded genes often enhance bacterial fitness (lysogenic conversion) and provide immunity against lytic phages via superinfection immunity [1,48,49,50]. The phylum Bacteroidetes is a diverse group of bacteria, which inhabit various environments, from glaciers to the human gut [51,52]. Some bacteria in this group are important pathogens, such as the fish pathogens *F. psychrophilum*, *F. branchiophilum*, and *F. columnare*. However, the knowledge of prophages in these bacteria has concentrated on *F. psychrophilum* [53]. Thus far, only lytic phages infecting *F. columnare* have been described [17,21]. To gain a more in-depth view of the genetic diversity and genome evolution within this species, prophage characterization is needed. Here, we identified a functional prophage inducing from a type strain ATCC 23463; comparisons with other genomic data suggest this phage type is widely spread among species belonging to the phylum Bacteroidetes.

The prophage fF4 was detected from the supernatant of strain ATCC 23463. The number of free phages in a culture was detected at levels of ~10^4^ PFU/mL and no clear effect of temperature was seen. There is an indication that fF4 is induced with mitomycin C but this should be studied in more detail. Morphologically, fF4 displays similarities with myoviruses. Genome sequencing revealed a dsDNA genome of ~32 kbp in length. It shares genome synteny with the transposable phages classified under Saltoviridae, especially displaying Mu-like genomic organization [44]. Further characterization is required to confirm whether fF4 could represent a new group of transposable phages and if it could be included in the Saltoviridae. From the four conserved proteins (GemA, Mor, portal protein, and DDE transposase) found in all Saltoviridae phages identified thus far, GemA was not identified in fF4-like phages. Overall, the identified genes were poorly conserved even at the amino acid level. The genome size of most transposable phages described so far is approximately 35–39 kbp [54], whereas the genome of fF4 is shorter (similarly to Vibrio phage Martha 12B12 with ~33 kb). Furthermore, we identified fF4-like putative prophages in several bacterial species, all belonging to the phylum Bacteroidetes. Conserved proteins (also typical of transposable phages) were found (see in Table 1 and Figure 3): DDE recombinase, the associated ATPase, and portal-associated proteins (DUF935 portal protein, DUF1320). In principle, they all followed the same genome organization. However, in some cases, the tail tape measure protein was located more to the right end of the genome. In most of these putative prophages, the genome size was near to the size of the fF4 genome (32 kbp); however, a few larger ones were also identified. Overall, transposable phages are widespread in bacterial genomes and they cause reorganization in their host genome. Whether the genetic diversity among *F. columnare* strains has been partly the result of a transposable phage needs further research on phage sequences in the *F. columnare* genomes.

Hulo et al. [43] mentioned that they performed a survey on partially assembled genomes that contained a transposable prophage, showing that the ends of the phage genomes are, in many cases, missing, while all internal fragments form a single contig. They suggested that if the prophage could be readily identified, possible genome heterogeneity and assembly problems could be identified conveniently. The recognition of fF4-type phages could also aid in assembling Bacteroidetes genomes, as many of the identified prophage sequences (Table 1) were located in the ends of whole-genome shotgun contigs.

We have shown previously that the infection patterns of the *F. columnare* phages are genotype-specific [17]. Here, the prophage fF4, which was initially detected from the supernatant of strain ATCC 23463, was able to replicate in four bacterial strains representing different genotypes (C, G, and E). Interestingly, the infection ability was linked with bacterial geographical origin; all sensitive strains originated from Northern Finland [17,24,55]. One possible explanation for this could be the lack of superinfection immunity (provided by similar existing prophages) in these strains. It is also possible that phage-host interaction is sensitive to temperature and strains adapted to colder temperatures have not gained immunity against this prophage. However, further studies are required to explain this peculiarity.

The role of CRISPR–Cas immunity is widely recognized as an important player in bacteria–phage coevolution. It can also provide fingerprints of past interactions between the host and phage. Since the majority of bacteria carrying temperate phages also carry CRISPR systems [8], mapping CRISPR spacers to prophage genomes can provide knowledge on the temporal and spatial aspects of the phage–bacterium interactions (e.g., [56]). In this study, the CRISPR array of the host bacterium ATCC 23463 revealed one spacer that was identical to the prophage genome in the RNA-targeting VI-B locus. We have previously observed phage targeting spacers in *F. columnare* strains that do not provide immunity against phages [21]. Whether this is result of anti-CRISPR proteins in the phage genomes or is related to an unfunctional interference phase is still unknown. The spacer matching fF4 terminase in the VI-B locus in Chinese strains Pf1 and TC1691 could also indicate that these strains have interacted with fF4 or a similar phage in the past. In addition, analysis of the putative prophage of strain ATCC 49512 (pp49512) and spacer sequences from complete genomes of *F. columnare* strains revealed past interactions with the prophage, despite the genetic type or geographical origin of the strain. Several unique spacers in different strains suggests past interaction with the prophage, rather than the conserved nature of the spacer sequences. Interestingly, the spacer sequences from the Type II-C locus were identical or contained only few mismatches compared with pp49512, while the spacers in the VI-B locus contained a high number of mismatches and could not be reliably assigned to match the prophage region (see Table S5). However, these results may indicate that the RNA-targeting CRISPR defense of the Type VI-B system may cause stronger evolutionary pressure for phage evolution via interfering with the expression of the phage genes.

The advantages of lysogeny have been detected in many bacterial species, especially in pathogenic bacteria [48]. While we did not identify any clear host-benefitting genes in fF4, it is plausible that the genetic material transferred by prophages has affected virulence in *F. columnare*. In addition, the wide geographical distribution of the prophage indicates a common evolutionary origin, which may have also influenced the genetic diversity of the host strains. The CRISPR spacers found from different hosts originating from different geographic origins exemplify this. Altogether, our work exemplifies the need for characterization of phage isolates in order to identify prophage sequences in bacterial genomes. This work also enables the identification of related prophages, especially from Bacteroidetes bacteria. To understand bacterial genome sequencing better, the identification of prophage sequences is vital.

## Figures and Tables

**Table 1:** The presence of fF4-like prophage in bacterial strains and species based on partial amino acid similarities combined with genome synteny (see also Table S4).

Genome/Contig with fF4-like ORFs in Similar Order	Accession Number	Nucleotide Coordinates of the Putative Prophage (Start of First ORF–End of Last ORF)
*Flavobacterium columnare* B185	NZ_CP010992.1	608,417–576,055
*Flavobacterium columnare* 94-081	NZ_CP013992.1	1,262,320–1,294,784
*Flavobacterium columnare* isolate CIP109753, whole genome shotgun sequence	NZ_OLKH00000000.1	102,261–71,112
*Flavobacterium psychrophilum* strain DK150	NZ_FYCB01000051.1	52,369–85,910
*Flavobacterium* sp. XS-5 chromosome	NZ_CP042831.1	3,593,721–3,559,120
*Flavobacterium* sp. 1E403 Scaffold15_1	NZ_SBII01000016.1	10,719–41,799
*Flagellimonas* sp. XY-359 Scaffold1	NZ_SRXX01000001.1	237,270–267,609
*Chryseobacterium hispanicum* strain KCTC 22104 CONTIG0010	NZ_QNUG01000010.1	15,024–49,549
Candidatus *Chryseobacterium massiliae* strain CCUG 51329 CONTIG024	NZ_QNVU01000024.1	7428–40,244
*Elizabethkingia occulta* strain F8124 F8124_contig_3	WP_108721221.1	248,884–216,243
*Pedobacter* sp. isolate Bin_56_2 c_000000055685	SSFR01000008.1	116,867–85,896
*Alistipes* sp. AF14-19 AF14-19.Scaf10,	NZ_QTXM01000010.1	76,089–107,924
*Chryseobacterium haifense* strain F4391 F4391_contig_48_consensus	NZ_RJTY01000066.1	40,400–5446
*Flavobacterium frigoris* strain DSM 15719	NZ_FOFZ01000007.1	33,154–67,468
*Flavobacterium aquicola* strain DSM 100880 Ga0196849_103	NZ_QUNI01000003.1	281,946–248,059
*Ornithobacterium rhinotracheale* DSM 15997	CP003283.1	2,304,714–2,272,508
*Bergeyella zoohelcum* strain NCTC11661	NZ_UFTJ01000005.1	39,475–5420
*Mangrovibacterium marinum* strain DSM 28823 Ga0183469_111	NZ_QAAD01000011.1	122,619–90,830
Parabacteroides sp. AF48-14 AF48-14.Scaf9	NZ_QUDI01000009	38,604–77,918
**Genomes/Contigs with Incomplete Prophage Areas**		
*Flavobacterium columnare* strain CF1 NODE_7	NZ_MTDC00000000.1	8103–36,689 (end of contig: 36,689)
*Flavobacterium columnare* strain 1215 NODE_70	NZ_MTCZ01000070	complete contig (length: 15,133)
*Flavobacterium columnare* strain 1201 NODE_26	NZ_MTCX01000026	2470–30,169
*Flavobacterium branchiophilum* NBRC 15030 = ATCC 35035 strain NBRC 15030 sequence028	NZ_BJXD01000028.1	31,317–54 (start of contig: 0)
*Aquimarina latercula* DSM 2041 H526DRAFT_scaffold00005.5	NZ_KE387186.1	384,870–404,541 (end of contig: 404,645)
*Aquimarina latercula* DSM 2041 H526DRAFT_scaffold00019.19_C	NZ_AUMK01000022.1	19,776–20,674 (end of contig: 20,778)
*Pedobacter* sp. isolate Bin_56_2 c_000000035689	SSFR01000019.1	60,143–85,295 (end of contig: 86,005)

## Supplementary Materials

The following are available online at https://www.mdpi.com/2076-2607/8/12/1919/s1. Figure S1: Alignments of the *Flavobacterium columnare* phage fF4 and the *F. columnare* strains CF1, 1215 and 1201 genome contig areas containing the partial phage sequence; Table S1: Bacterial strains used for testing the fF4 host range; Table S2: Primers used for connecting the three contigs of the fF4 genome; Table S3: BlastP and HHPred analysis of fF4 open reading frames; Table S4: Similarities among the putative fF4-like prophages presented by the ORF number in each of the genome regions analyzed; Table S5: Prophage search using PHASTER and VirSorter; Table S6: Analysis of pp49512; Table S7: CRISPR spacer hits in the genomic area of ATCC49512 with putative prophage pp49512.
